# Mobilizable Plasmids for Tunable Gene Expression in *Francisella novicida*

**DOI:** 10.3389/fcimb.2018.00284

**Published:** 2018-08-31

**Authors:** Maj Brodmann, Rosalie Heilig, Petr Broz, Marek Basler

**Affiliations:** ^1^Biozentrum, University of Basel, Basel, Switzerland; ^2^Department of Biochemistry, University of Lausanne, Épalinges, Switzerland

**Keywords:** *Francisella novicida*, expression plasmid, conjugation, ATc inducible, complementation, type VI secretion system, bacterial mutagenesis, tularemia

## Abstract

*Francisella tularensis* is the causative agent of the life-threatening disease tularemia. However, the molecular tools to study *Francisella* are limited. Especially, expression plasmids are sparse and difficult to use, as they are unstable and prone to spontaneous loss. Most *Francisella* expression plasmids lack inducible promoters making it difficult to control gene expression levels. In addition, available expression plasmids are mainly designed for *F. tularensis*, however, genetic differences including restriction-modification systems impede the use of these plasmids in *F. novicida*, which is often used as a model organism to study *Francisella* pathogenesis. Here we report construction and characterization of two mobilizable plasmids (pFNMB1 and pFNMB2) designed for regulated gene expression in *F. novicida*. pFNMB plasmids contain a tetracycline inducible promoter to control gene expression levels and *oriT* for RP4 mediated mobilization. We show that both plasmids are stably maintained in bacteria for more than 40 generations over 4 days of culturing in the absence of selection against plasmid loss. Expression levels are dependent on anhydrotetracycline concentration and homogeneous in a bacterial population. pFNMB1 and pFNMB2 plasmids differ in the sequence between promoter and translation start site and thus allow to reach different maximum levels of protein expression. We used pFNMB1 and pFNMB2 for complementation of *Francisella* Pathogenicity Island mutants Δ*iglF*, Δ*iglI*, and Δ*iglC in-vitro* and pFNMB1 to complement Δ*iglI* mutant in bone marrow derived macrophages.

## Introduction

*Francisella tularensis* is the causative agent of tularemia and can cause life-threatening disease in animals and humans. Essential for *Francisella* virulence is the *Francisella* pathogenicity island (FPI), which encodes a dynamic type VI secretion system (T6SS) (Bröms et al., 2010; Chong and Celli, 2010; Clemens et al., 2015; Brodmann et al., 2017). The most virulent subspecies *F. tularensis* subspecies *tularensis*, classified as a Tier 1 agent (Oyston et al., 2004), and subspecies *holarctica* (hereafter *F. tularensis*) contain two FPIs. The related subspecies *F. tularensis* subspecies *novicida* (hereafter *F. novicida*) possesses only one FPI and is highly virulent in mice but rarely infects humans. These features make *F. novicida* an ideal model organism for investigating tularemia and *Francisella* T6SS (Kingry and Petersen, 2014).

Molecular tools to make chromosomal in-frame deletion mutations in *Francisella* are available and commonly used to study the role of a certain gene of interest on a particular phenotype (Anthony et al., 1991; Golovliov et al., 2003; Frank and Zahrt, 2007). However, gene deletion and insertions may alter the expression of neighboring genes and cause so called “polar effects”. If expression of the gene of interest *in trans* from an inducible plasmid reverses the mutant phenotype, a possible polar effect can be ruled out. Unfortunately, only few expression plasmids are available for *Francisella*. Therefore, many recent studies lack *in trans* complementation of in-frame deletion mutations (Nano and Schmerk, 2007; Santic et al., 2011; Eshraghi et al., 2016; Brodmann et al., 2017) or use chromosomal complementation *in cis* (de Bruin et al., 2007; Weiss et al., 2007; Lindgren et al., 2013).

**Figure 1:**
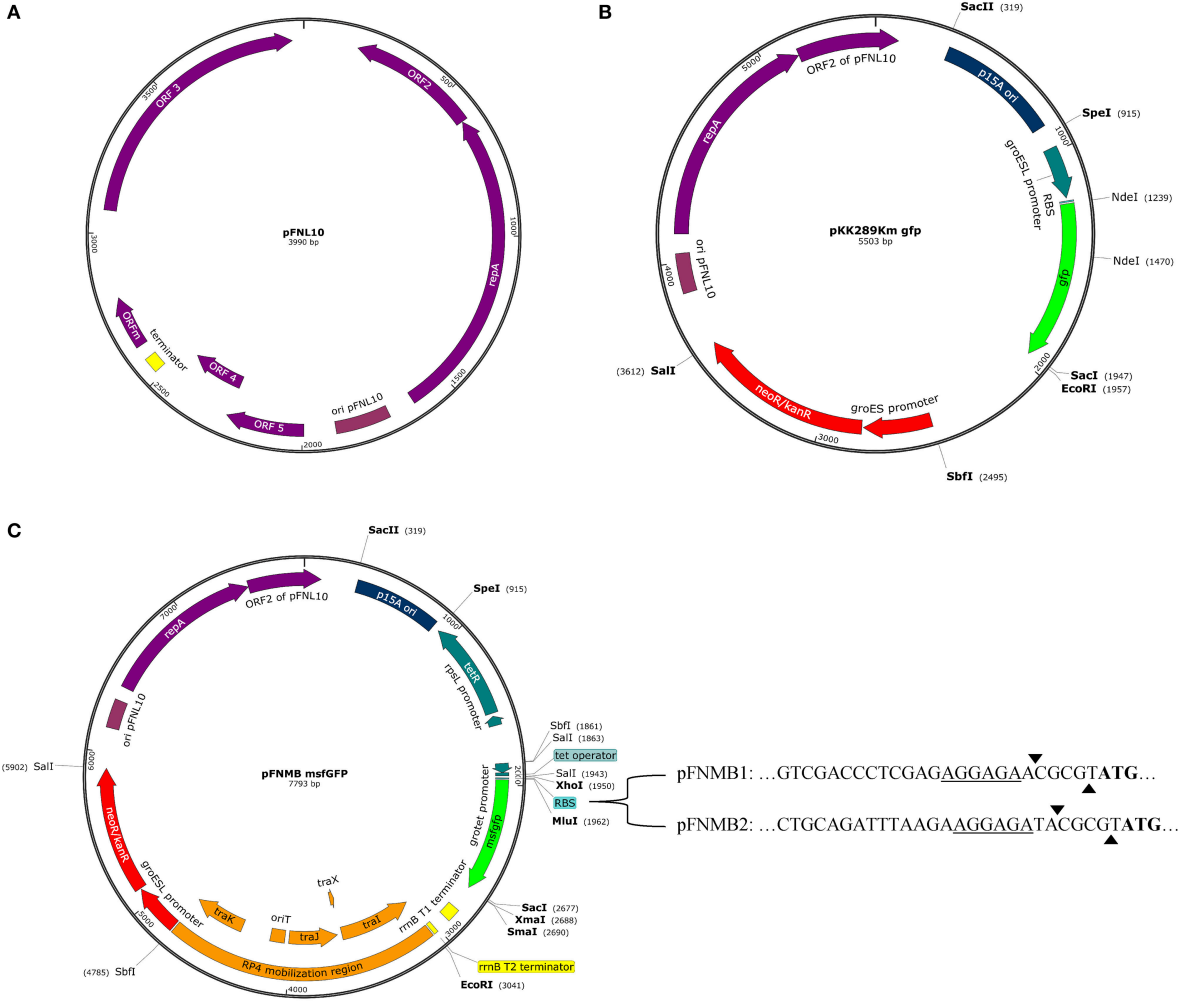
Plasmid maps. Promoters, genes, terminators, and some relevant restriction sites are shown. Maps were designed with SnapGene Version 4.0.3. **(A)** Plasmid map of pFNL10 according to Pomerantsev et al. (2001a). **(B)** Plasmid map of pKK289Km *gfp* according to Bönquist et al. (2008). **(C)** Plasmid map of pFNMB *msfgfp* with variable RBS sequences. pFNMB1 contains the RBS sequence of *iglC* and pFNMB2 of pKK289Km. The underline highlights the RBS, the arrows mark the MluI restriction site and the start codon is represented in bold.

All available expression plasmids for *Francisella* are derived from the pFNL10 plasmid except for pCUG18, which is derived from pC194 and pUC18 (Rasko et al., 2007). pFNL10 was isolated from the *F. novicida*-like strain F6168 (Pavlov et al., 1996). The function of pFNL10 is unclear; however, the five encoding regions on the plasmid were identified. ORF1—ORF3 are required for plasmid replication and encode replication initiation protein RepA (ORF1), an ATP-dependent RNA helicase/endonuclease (ORF2), and an integrase/recombinase (ORF3). ORF4 and ORF5 encode a putative toxin-antitoxin system together with a possible regulatory feature ORFm (Pomerantsev et al., 2001a) (Figure 1A). Over the last 20 years, pFNL10 was modified to meet the needs of the *Francisella* research community. First, tetracycline and chloramphenicol resistance cassettes were introduced for selection resulting in pFNL200 (Pavlov et al., 1996). Since pFNL200 was restricted to replicate in *Francisella*, the p15A origin of replication of *Escherichia coli* was added thus obtaining a shuttle vector pKK202 (Norqvist et al., 1996). Later, the constitutively active *gro*ESL promoter was successfully used to express *gfp* and other genes (pKK214, pKK289Km, Figure 1B) *in-vitro* and in eukaryotic cells (Abd et al., 2003; Bönquist et al., 2008). Other pFNL10 derivatives are pFNLTP, which includes a version that only replicates at 32°C but not at 42°C due to a mutation in *repA* (Maier et al., 2004) and pMP, which includes a version of a *bla* promoter that is not recognized in *E. coli* to allow cloning of toxic genes in *E. coli* (LoVullo et al., 2006, 2009). So far, only two controllable *Francisella* promoter systems exist; a glucose repressible system (pTCD3) (Horzempa et al., 2008) and a tetracycline inducible or repressible version of the *gro*ESL promoter (pEDL) (LoVullo et al., 2012). The tetracycline inducible promoter system is a preferred choice for many bacterial model organisms because it allows tight and concentration dependent regulation of expression levels. It is also applicable for infection models such as cell cultures or animals since tetracycline passively penetrates most mammalian membranes (Bertram and Hillen, 2008). The tetracycline inducible promoter systems consists of constitutively expressed TetR, which binds to the *tetO* sequence and thereby transcriptionally represses the *tetA* promoter. Tetracycline or anhydrotetracycline (ATc), which is less toxic but has even higher affinity to TetR, binds TetR, and derepresses the *tetA* promoter (Gossen and Bujard, 1992). In the case of the tetracycline repressible promoter system, TetR binds *tetO* only if tetracycline or ATc is present, therefore, transcription is repressed upon addition of ATc (Scholz et al., 2004).

Despite the efforts in recent years, complementation from plasmid remains difficult in *Francisella*. Non-native expression levels (Santic et al., 2007; Zogaj and Klose, 2010) and spontaneous deletions in pFNL200 (Pomerantsev et al., 2001b) and pFNLTP (Maier et al., 2004) were reported. Another problem is the relatively low electroporation efficiency in *Francisella* and especially in *F. novicida* for plasmids isolated from *E. coli*. This is thought to be due to active restriction-modification systems in *Francisella* (Maier et al., 2004; LoVullo et al., 2006). In *F. novicida*, 4 restriction-modification systems were identified to restrict unmodified plasmid DNA, while in *F. tularensis* most restriction-modification system were annotated as pseudogenes (Gallagher et al., 2008). Expression plasmids were mainly tested in *F. tularensis* (Norqvist et al., 1996; Abd et al., 2003; LoVullo et al., 2006, 2009, 2012; Rasko et al., 2007) and consequently, in-frame deletions were more often complemented from plasmid in *F. tularensis* (Lai et al., 2004; Gil et al., 2006; Maier et al., 2006; Bönquist et al., 2008; Ark and Mann, 2011; Lindemann et al., 2011; Schmidt et al., 2013). On the other hand, suitable expression plasmids are mostly lacking in *F. novicida* and therefore only few studies include complementation experiments (Tempel et al., 2006; de Bruin et al., 2011).

Here we report construction of expression plasmids derived from pKK289Km specially designed for *F. novicida*. pFNMB1 and pFNMB2 plasmids can be mobilized by conjugation to overcome the need for electroporation. In addition, ATc induction allows homogeneous gene expression and the plasmids are stably maintained in a population for 4 days without selection pressure. As a proof of concept, we successfully complemented in-frame deletion of FPI genes *iglF, iglI*, and *iglC in-vitro* and *iglI* in bone marrow derived macrophages.

## Materials and methods

### Bacterial strains and growth conditions

*Francisella tularensis* subsp. *novicida* strain U112 and the derivative strains were grown in brain heart infusion (BHI) broth supplemented with 0.2% L-cysteine (Sigma). Ampicillin (100 μg/ml, AppliChem) or kanamycin (15 μg/ml, AppliChem) were added if not stated otherwise. Liquid cultures were grown aerobically at 37°C. Gene expression from plasmid was induced by adding the indicated concentration of anhydrotetracycline (ATc, IBA) to the liquid culture at OD_600_ of 0.02 for 3 h. *Escherichia coli* DH5α λ*pir* and derivative strains were grown aerobically in Luria broth (LB) or on LB agar plates both supplemented with 50 μg/ml kanamycin at 37°C. All strains used are listed in Supplementary Table 1.

### Construction of plasmids

All plasmids and corresponding primers are listed in Supplementary Table 2. Expression plasmids pFNMB1 and pFNMB2 were constructed by using the backbone of pKK289Km *gfp* (Bönquist et al., 2008) and inserting the RP4 mobilization site of pDMK3 (Lindgren et al., 2007) at EcoRI and SbfI restriction sites, thereby removing the truncated chloramphenicol resistance cassette, a leftover of pKK214CAT (Abd et al., 2003). Then the ATc inducible promoter cassette of pEDL17 (*tetR* with *rpsL* promoter and *groESL* promoter with *tetO*; LoVullo et al., 2012), the multiple cloning site of pDMK3 and the *E. coli rrnB* T1 and T2 terminators of pBAD24 (Guzman et al., 1995) were combined by overlap-extension PCR. The PCR product was placed into pKK289Km *gfp* at SpeI and EcoRI restriction sites to remove the original *groESL* promoter and *gfp* gene. For pFNMB1, the *iglC* RBS was inserted together with *msfgfp* at XhoI and XmaI restriction sites by amplification of *msfgfp* with primers containing the sequence of *iglC* RBS and an additional MluI restriction site in front of the *msfgfp* start codon (AGAGGAGAACGCGT). For pFNMB2, the *iglC* RBS was exchanged for the RBS of pKK289Km by combining the ATc inducible promoter cassette of pEDL17 and *msfgfp* by overlap-extension PCR using primers containing the RBS of pKK289Km with a MluI restriction site. The PCR product was placed into pFNMB1 at SpeI and XmaI restriction sites. All cloning products were sequenced. Plasmid maps were generated with SnapGene Version 4.0.3. pFNMB1 *msfgfp* (Addgene ID: 113191) and pFNMB2 *msfgfp* (Addgene ID: 113192) were deposited to Addgene.

### Bacterial mutagenesis

Suicide vector pDMK3 was used for generating in-frame deletions as reported previously (Lindgren et al., 2007; Brodmann et al., 2017), except that an optimized conjugation procedure was used (described below). Various genes were cloned into pKK289Km using NdeI and EcoRI or SacI restriction sites and into pFNMB1 and pFNMB2 using MluI and SacI restriction sites. Plasmids, remaining peptides of in-frame deletions and primers are listed in Supplementary Table 3. Cloning products were sequenced and the site of homologous recombination was verified by PCR with primers located outside of the replaced regions.

pKK289Km and derivatives were transformed by electroporation as reported previously (Maier et al., 2004). Up to 1 μg of plasmid was used for electroporation. pFNMB1, pFNMB2 and derivatives were mobilized by conjugation as described below.

### Conjugation

*F. novicida* was grown on BHI agar plates supplemented with 0.2 % L-cysteine and 100 μg/ml ampicillin and the donor *E. coli* strain (kind gift of A. Harms and C. Dehio, Harms et al., 2017) harboring the plasmid of interest was grown on Luria-Bertani (LB) agar plates supplemented with 300 μM 2,6-Diaminopimelic acid (Sigma) and 50 μg/ml kanamycin. Both plates were incubated over night at 37°C. The following day, about 100 μl of *F. novicida* and *E. coli* dense bacterial cultures were transferred to a fresh LB plate supplemented with 300 μM 2,6-Diaminopimelic acid and mixed thoroughly. After 2 h incubation at 37°C, about 50 μl of the mixture was resuspended in 100 μl Mueller–Hinton (MH) broth and plated on MH agar plates supplemented with 0.1% L-cysteine, 0.1% D-glucose (Millipore), 0.1% FCS (BioConcept), 100 μg/ml ampicillin, and 15 μg/ml kanamycin and incubated for 2 days at 37°C. Single *F. novicida* colonies were purified by passaging on selective plates.

To assess conjugation efficiency, the donor and recipient strains were first concentrated to an OD_600_ of 10 and then mixed in a 1 to 1 ratio (each 50 μl). Five microliters of the mixture was spotted on a LB agar plate supplemented with 300 μM 2,6-Diaminopimelic acid in two technical replicates. After 2 h, the spots were cut out and resuspended in 100 μl of MH broth. The resuspended bacteria were plated on MH agar plates supplemented with 0.1% L-cysteine, 0.1% D-glucose, 0.1% FCS, 100 μg/ml ampicillin, and 15 μg/ml kanamycin. The CFU per ml and the conjugation efficiency were calculated in the following manner:

$$\begin{array}{l} Transformants\;\left(\frac{CFU}{ml}\right)=\;\frac{average\;colonies_{counted}}{0.1\;ml} \end{array}$$

$$\begin{array}{l} Conjugation\;efficency=\frac{calculated\;transformants\;}{used\;donor\;cells} \end{array}$$

The assay was performed in three biological replicates.

### Plasmid stability assay

On day 0, *F. novicida* harboring pFNMB1 *msfgfp* or pFNMB2 *msfgfp* were diluted to an OD_600_ of 0.02 and grown in liquid overnight (ON) cultures supplemented with 15 μg/ml kanamycin and 500 ng/ml ATc to induce gene expression. On days 1–4, the old ON cultures were diluted to an OD_600_ of 0.02 and supplemented with 100 μg/ml ampicillin and 500 ng/ml ATc. For every ON culture, OD_600_ was measured and aliquots were taken for imaging, serial dilutions and inoculation of new ON cultures. Serial dilutions were plated on MH agar plates supplemented with 0.1% L-cysteine, 0.1% D-glucose, 0.1% FCS, and 100 μg/ml ampicillin and on MH agar plates supplemented with 0.1% L-cysteine, 0.1% D-glucose, 0.1% FCS, and 100 μg/ml ampicillin and 15 μg/ml kanamycin. Colony forming units (CFU) were counted and the concentrations of CFU/ml were calculated. Number of generations were calculated with following formula:

$$\begin{array}{l} N_{0}=calculated\;concentration\;of\;bacteria\;used\;for\;inoculation \end{array}$$

$$\begin{array}{l} N=calculated\;concentration\;of\;bacteria\;after\;serial\;dilution \end{array}$$

$$\begin{array}{l} Number\;of\;generations\;n=\frac{\log\frac{N}{N_{0}}}{\log2} \end{array}$$

The experiment was carried out in three biological replicates.

### Plasmid recovery

pFNMB1 *msfgfp* and pFNMB2 *msfgfp* were recovered from *F. novicida* with a Zyppy Plasmid Miniprep Kit (Zymo Research) after passaging the cultures for 4 days in liquid BHI supplemented with 100 μg/ml ampicillin and 500 ng/ml ATc as described above. About 250 ng of each plasmid DNA was then transformed into chemo-competent *E. coli* DH5α λ*pir*. The transformed *E. coli* were plated on LB agar plates supplemented with 50 μg/ml kanamycin. Three independent experiments were carried out. The next day, colonies were grown in liquid LB supplemented with 50 μg/ml kanamycin, plasmid DNA was isolated and 250 ng of each plasmid was digested with SacI-HF and SpeI restriction enzymes (New England BioLabs) for 1 h. As control, both plasmids were additionally isolated from *E. coli* directly, without passaging in *F. novicida*, and digested identically. After heat inactivation of the enzymes (80°C for 20 min), the digested plasmids were loaded on a 1% agarose gel (BioConcept) together with a 1 kb ladder (New England BioLabs). DNA was stained with RedSafe (iNtRON Biotechnology) and a Red imaging system (Alpha Innotech) was used for imaging.

### Fluorescence microscopy

Microscope set up was described previously (Kudryashev et al., 2015; Vettiger and Basler, 2016; Brodmann et al., 2017). *F. novicida* strains were prepared as described in Brodmann et al. (2017). For assessment of plasmid stability, 1.5 μl ON culture was spotted on a pad of 1% agarose in phosphate buffered saline (PBS) and imaged immediately. For measuring the GFP signal intensities after induction with ATc, the spotted bacteria were imaged immediately. For assessing T6SS function of complemented in-frame deletion mutants, the bacteria were incubated on a pad at 37°C for 1 h before imaging. All imaging experiments were performed in three independent experiments.

### Image analysis

Image analysis and manipulations were performed with Fiji software (Schindelin et al., 2012) as described previously (Basler et al., 2013; Vettiger and Basler, 2016). For calculation of the GFP signal intensities after ATc induction, the background intensity was subtracted with the plugin “BackgroundSubstracter.” Then the plugin “Time Series Analyzer V3.0” was used to quantify the total GFP signal intensity of the whole field of view. The total GFP signal intensity was divided by the number of bacteria in the field of view. Number of bacteria was calculated with the “Find Maxima” function from phase contrast images. Contrast on compared images was adjusted equally. For the Supplementary Movies, the contrast used for *F. novicida* U112 Δ*iglC* pFNMB2 *iglC* induced with 500 ng/ml was set to match the other strains.

### Cell culture and infection assay

The day before infection experiment, bone marrow derived macrophages (BMDMs) were seeded into 96-well plates (Eppendorf) at a density of 5^*^10^4^ cells/well in DMEM (Thermo Fisher) with 20% M-CSF (supernatant of L929 mouse fibroblasts, BioConcept), 10% FCS (BioConcept), 10 mM HEPES (BioConcept), and non-essential amino acids (Thermo Fisher). The BMDMs were primed with 100 ng/ml LPS from *E. coli* O111:B4 (InvivoGen). *F. novicida* strains were grown aerobically in liquid BHI culture supplemented with the corresponding antibiotics and with 0 or 500 ng/ml ATc at 37°C ON. The next day, the medium of the BMDMs was replaced with fresh medium supplemented with 0 or 1,000 ng/ml ATc and the bacteria were added to the BMDMs at a multiplicity of infection (MOI) of 100. The 96-well plates were centrifuged at 300 g for 5 min to synchronize the infection process and afterwards incubated at 37°C. After 2 h, the medium was replaced with fresh medium supplemented with 0 or 1,000 ng/ml ATC and with 10 μg/ml gentamycin (BioConcept). Then the 96-well plates were incubated for 10 h at 37°C. Afterwards, a lactate dehydrogenase (LDH) release assay was carried out with an LDH Cytotoxicity Detection Kit (Takara). The percentage of LDH release was calculated with the following formula:

$$\begin{array}{l} \%\;of\;LDH\;release=\frac{{LDH\;value}_{infected}-{LDH\;value\;}_{uninfected}}{{LDH\;value}_{total\;lysis}-{LDH\;value\;}_{uninfected}}\times 100 \end{array}$$

Infection experiments were carried out in biological triplicates. The unpaired two-tailed *t-*test with Welch's correction was used to identify significant differences. *P*-values are given in the figure legend.

## Results

The need for expression plasmids for *F*. *novicida*, motivated us to construct the mobilizable and inducible expression plasmids pFNMB1 and pFNMB2 (Figure 1C). We constructed pFNMB1 and pFNMB2 by using the backbone of pKK289Km, which is transformed by electroporation and contains a constitutively active promoter *gro*ESL (Bönquist et al., 2008). As electroporation can be difficult in *F. novicida* (Maier et al., 2004; LoVullo et al., 2006), the need for electroporation was circumvented by inserting the RP4 mobilization site of pDMK3 (Lindgren et al., 2013) encoding *traI* (relaxase), *traX* (regulation of *traI* and *traJ*), *traJ*, and *traK* (*oriT* binding proteins) and origin of transfer (*oriT*) (Haase et al., 1995) at the site of the truncated chloramphenicol resistance cassette. The constitutively active *gro*ESL promoter was exchanged for the tetracycline inducible *grotet* promoter (LoVullo et al., 2012). Two different RBS were inserted to achieve a wider range of expression levels. pFNMB1 was designed for lower expression and contains the ribosomal binding site (RBS) of *iglC* in front of a MluI restriction site. Higher expression levels in pFNMB2 were reached by inserting the RBS of pKK289Km in front of a MluI restriction site. In addition, the well characterized *E. coli rrnB* T1 and T2 terminators from pBAD (Guzman et al., 1995) were inserted after a multiple cloning site.

First, we tested the conjugation efficiency of pFNMB1 *msfgfp* from an *E. coli* strain harboring a chromosomally encoded RP4 machinery (Harms et al., 2017) to *F. novicida*. Both strains were mixed in a 1:1 ratio and spotted on an agar plate. After 2 h incubation at 37°C, the bacteria were resuspended and plated on agar plates containing both ampicillin and kanamycin to select for *F. novicida* harboring the plasmid. On average, about 5.1^*^10^−7^ ± 2.5^*^10^−7^ bacterial cells were transformed per donor cell.

**Figure 2:**
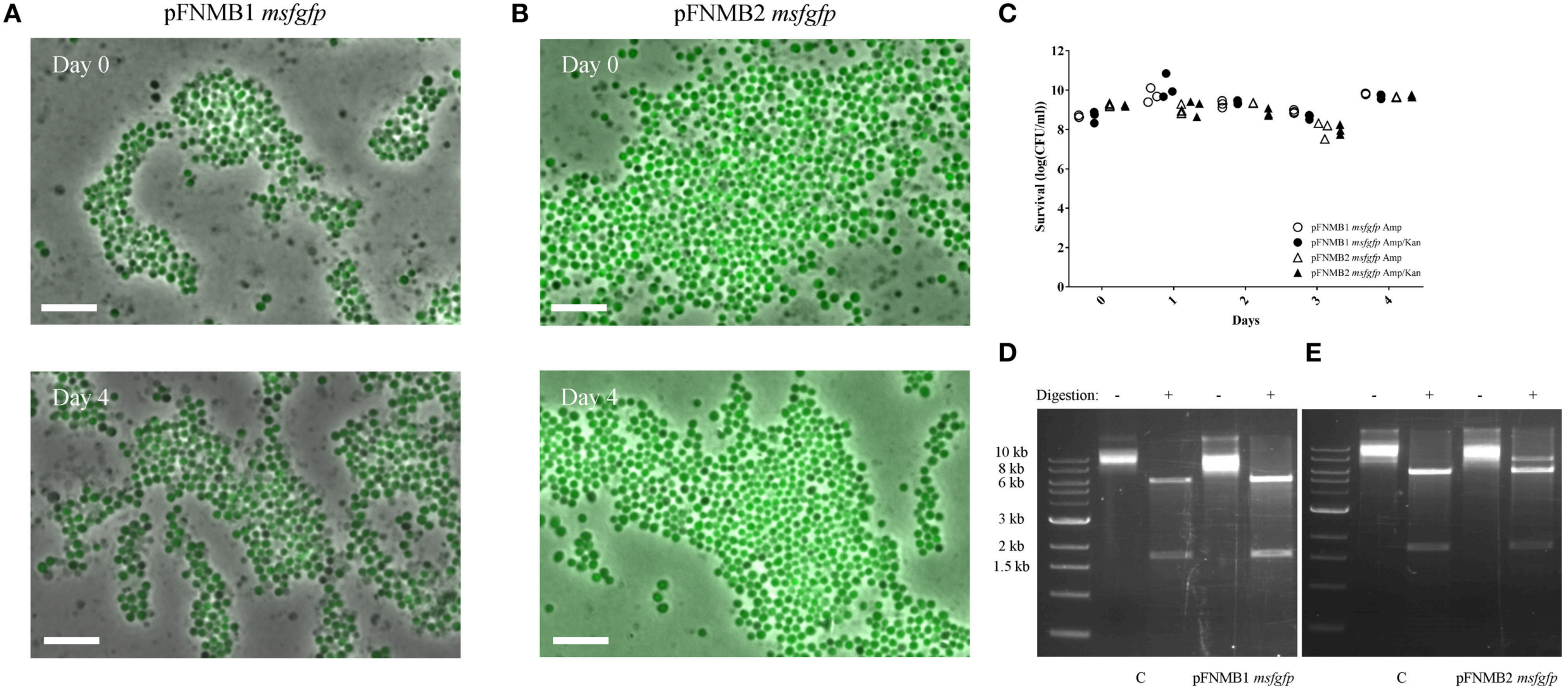
Plasmid stability. GFP expression was induced with 500 ng/ml of ATc. ON culture of day 0 was supplemented with 15 μg/ml kanamycin, while ON cultures of days 1–4 were supplemented with 50 μg/ml ampicillin. **(A)** GFP expression in *F. novicida* U112 pFNMB1 *msfgfp* ON cultures of day 0 and day 4. **(B)** GFP expression in *F. novicida* U112 pFNMB2 *msfgfp* ON cultures of day 0 and day 4. **(A,B)** Images are a merge of phase contrast and GFP channel. 26 × 39 μm fields of view are shown. Scale bar represent 5 μm. Representative replicates are shown. Three independent experiments were performed. **(C)** Survival assay performed with ON cultures of *F. novicida* U112 pFNMB1 *msfgfp* and *F. novicida* U112 pFNMB2 *msfgfp* plated on ampicillin and ampicillin/kanamycin plates. Three independent experiments were performed. **(D)** Digestion of pFNMB1 *msfgfp* with SacI and SpeI restriction enzymes. **(E)** Digestion of pFNMB2 *msfgfp* with SacI and SpeI restriction enzymes. **(D,E)** Plasmids were passaged in *F. novicida* for 4 days before being transformed into *E. coli*. Controls were isolated directly from *E. coli*. Representative replicates are shown. Three independent experiments were performed.

As plasmid instability is reported for certain *Francisella* plasmids (Pomerantsev et al., 2001b; Maier et al., 2004), we tested the stability of pFNMB1 and pFNMB2 with *msfgfp* in *F. novicida* over 4 days by inducing expression with 500 ng/ml ATc but without addition of kanamycin to select for plasmid maintenance (Figure 2). To assess plasmid stability, we monitored msfGFP expression by fluorescence microscopy (Figures 2A,B) and counted the kanamycin resistant colonies (Figure 2C). Over 4 days and during ~40 generations, the plasmids were stable in the bacterial population. Importantly both, the *msfgfp* and the kanamycin resistance cassette, which are located at different sites on the plasmid (Figure 1C), stayed fully functional. To exclude that the plasmids integrated into the chromosome, pFNMB1 *msgfp* and pFNMB2 *msfgfp* were recovered from *F. novicida* after passaging the bacteria for 4 days as described above. Then the isolated plasmid DNA was transformed into *E. coli*. The plasmids were recovered again, digested with SacI and SpeI restriction enzymes and loaded on an agarose gel to analyze the size of the DNA fragments. Two bands of the correct size (about 6,000 base pairs and 1,700 base pairs) were observed for pFNMB1 *msfgfp* and pFNMB2 *msfgfp* similarly to the controls pFNMB1 *msfgfp* and pFNMB2 *msfgfp*, which were not passaged in *F. novicida* (Figures 2D,E). These results strongly suggest that the plasmids are maintained extra-chromosomally in *F. novicida* without any rearrangements.

**Figure 3:**
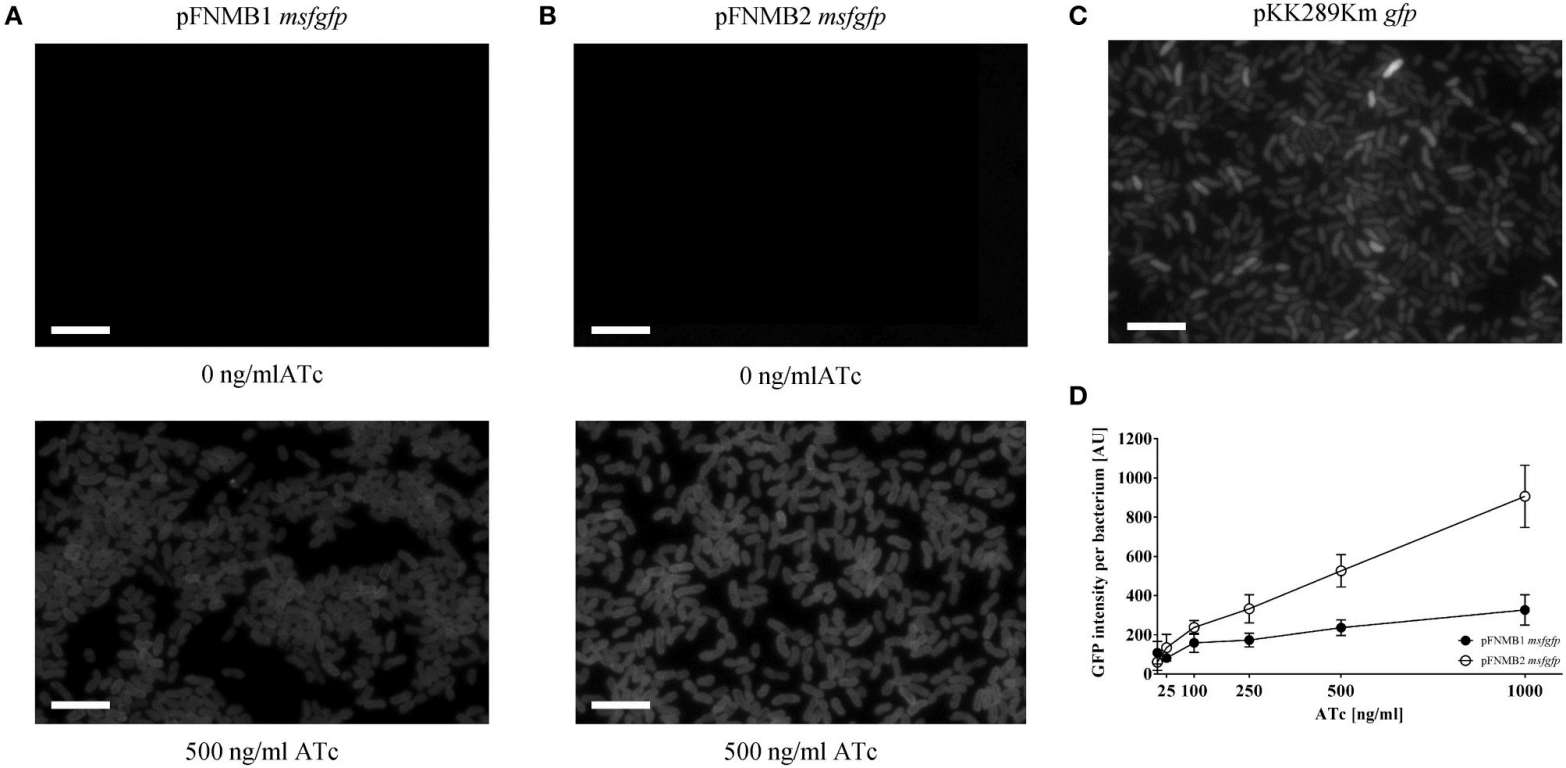
GFP intensities of pFNMB1 *msfgfp*, pFNMB2 *msfgfp*, and pKK289Km *gfp*. **(A)** Homogeneous GFP expression in *F. novicida* U112 pFNMB1 *msfgfp* upon induction with 500 ng/ml ATc. **(B)** Homogeneous GFP expression in *F. novicida* U112 pFNMB2 *msfgfp* upon induction with 500 ng/ml ATc. **(C)** Heterogeneous GFP expression in *F. novicida* U112 pKK289Km *gfp*. **(A–C)** GFP expression was induced with 0 ng/ml ATc and 500 ng/ml of ATc. GFP channels are shown. 39 × 26 μm fields of view. Scale bars represent 5 μm. **(D)** GFP expression in *F. novicida* U112 pFNMB1 *msfgfp* and *F. novicida* U112 pFNMB2 *msfgfp* was induced with 0, 25, 100, 250, 500, and 1,000 ng/ml of ATc. GFP intensity per bacterium was calculated as described in the section Materials and Methods. Three independent experiments were performed. Standard deviations are shown.

To test if the *grotet* promoters of pFNMB1 and pFNMB2 respond to ATc in *F. novicida*, we used different ATc concentrations to induce expression of msfGFP (Figure 3). Indeed, msfGFP intensity increased in a concentration dependent manner for both plasmids. However, the level of induction differed; GFP expression from pFNMB1 was in general lower than from pFNMB2 (Figure 3D) indicating that the pKK289Km RBS starts translation more efficiently than the *iglC* RBS. Furthermore, we compared GFP expression from pFNMB1, pFNMB2 and pKK289Km by fluorescence microscopy (Figures 3A–C). Interestingly, bacteria harboring pKK289Km *gfp* showed a heterogeneous expression of GFP (Figure 3C), while all bacteria harboring pFNMB1 *msfgfp* or pFNMB2 *msfgfp* expressed similar levels of GFP after induction with 500 ng/ml ATc. Without ATc, no GFP fluorescence was observed indicating that expression is well repressed by the TetR in the absence of ATc (Figures 3A,B). However, GFP expression was higher in some bacteria containing pKK289Km plasmid than in those with pFNMB1 and pFNMB2 (Figures 3A–C).

**Figure 4:**
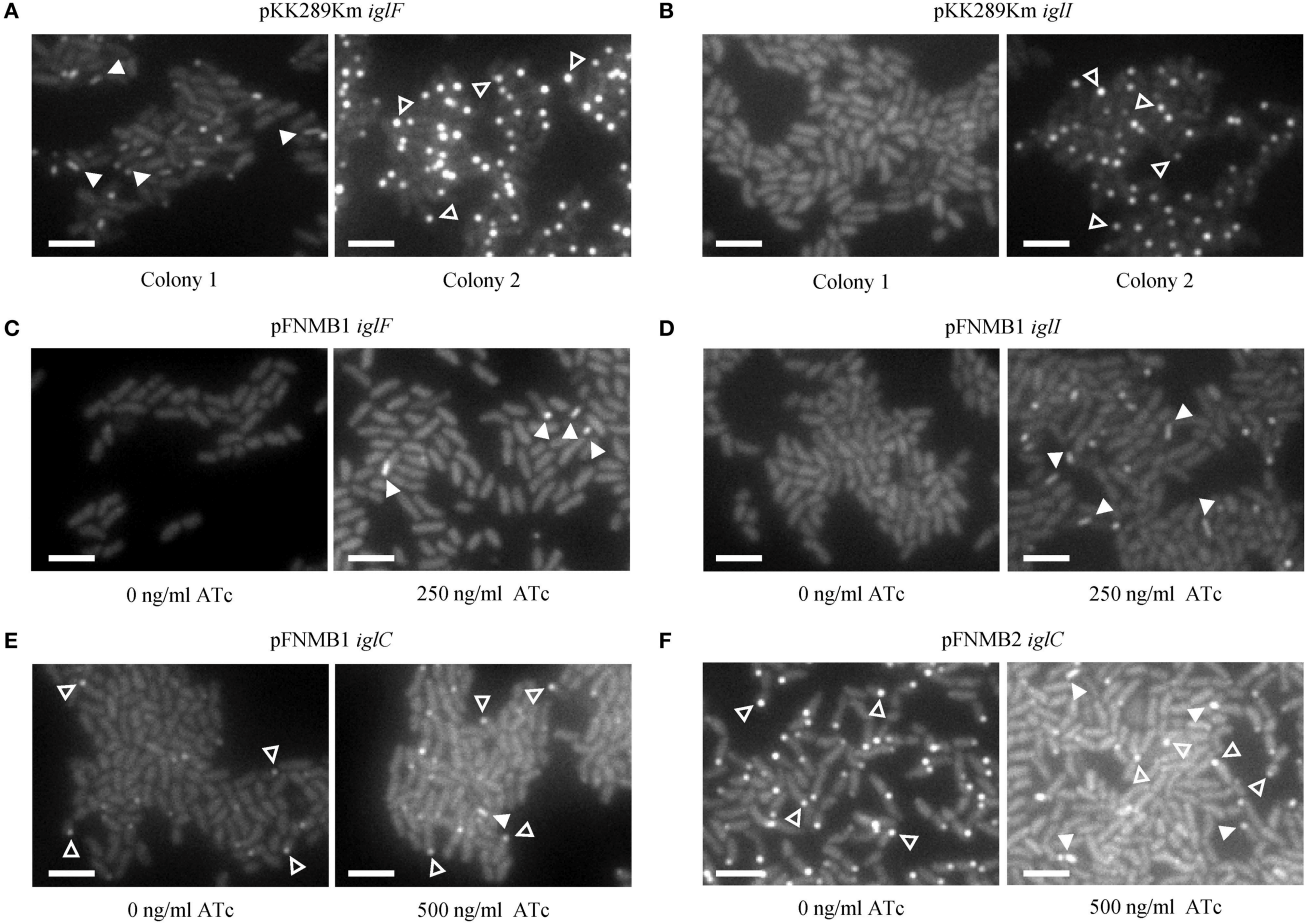
Complementation of in-frame deletion mutants on agar pads. The first frame of the GFP channel of one of the examples in the Supplementary Movie 2 is shown. T6SS assemblies were distinguished from GFP aggregates by analyzing dynamics in the movies. A maximum of 4 T6SS assemblies (arrow heads) and/or GFP aggregates (empty arrow heads) are highlighted. 13 × 9.75 μm fields of view. Scale bars represent 2 μm. **(A)** T6SS sheath assemblies (arrow heads) in *F. novicida* U112 *iglA-sfgfp* Δ*iglF* pKK289Km *iglF* colony 1 and GFP aggregates (empty arrow heads) in colony 2 **(B)** Failed T6SS sheath assembly in *F. novicida* U112 *iglA-sfgfp* Δ*iglI* pKK289Km *iglI* colony 1 and GFP aggregates (empty arrow heads) in colony 2. **(C)** T6SS sheath assemblies (arrow heads) in *F. novicida* U112 *iglA-sfgfp* Δ*iglF* pFNMB1 *iglF*, IglF expression induced with 250 ng/ml of ATc. **(D)** T6SS sheath assemblies (arrow heads) in *F. novicida* U112 *iglA-sfgfp* Δ*iglI* pFNMB1 *iglI*, IglI expression induced with 250 ng/ml of ATc. **(E)** One T6SS sheath assembly (arrow head) and GFP aggregates (empty arrow heads) in *F. novicida* U112 *iglA-sfgfp* Δ*iglC* pFNMB1 *iglC*, IglC expression induced with 500 ng/ml of ATc. **(F)** T6SS sheath assemblies (arrow heads) and GFP aggregates (empty arrow heads) in *F. novicida* U112 *iglA-sfgfp* Δ*iglC* pFNMB2 *iglC*, IglC expression induced with 500 ng/ml of ATc.

In our previous study, we constructed several in-frame deletion mutants in *F. novicida* and assessed T6SS function using fluorescence microscopy (Brodmann et al., 2017). For two mutants (Δ*iglF* and Δ*iglI*) with abolished T6SS function, we were unable to exclude polar effects as the deletion of the downstream genes (*iglG* and *iglJ*) resulted in similar phenotypes. Here, we generated *F. novicida* mutants carrying the respective complementation plasmids and successfully restored T6SS sheath assembly in Δ*iglF* and Δ*iglI* mutants by expression of IglF or IglI from pFNMB1 after induction with 250 ng/ml ATc (Figures 4C,D, Supplementary Movies 1, 2). Importantly, independently isolated colonies exhibited the same phenotypes. This was in contrast with several problems we experienced when using pKK289Km plasmid. First, electroporation of pKK289Km was very inefficient, as we routinely obtained only 1–10 transformed colonies even when using 1 μg of the plasmid DNA and 3^*^10^10^ *F. novicida* cells. In addition, independently isolated colonies exhibited different phenotypes such as no complementation, partial complementation or we only detected IglA-GFP aggregates in cells (Figures 4A,B, Supplementary Movies 1, 2) suggesting spontaneous deletions or variable expression levels. As previously characterized (Brodmann et al., 2017), T6SS dynamics in *F. novicida* consists of assembly, contraction and disassembly of a long cytosolic sheath at the bacterial poles and thus non-dynamic GFP aggregates likely represent non-functional T6SS (Supplementary Movies 1, 2). We also tested ATc inducible plasmid pEDL17 (LoVullo et al., 2012) for complementation, however, we failed to obtain any *F. novicida* colonies containing the plasmid.

To further test pFNMB1 and pFNMB2 plasmids, we attempted to restore T6SS function in a Δ*iglC* mutant. The IglC protein is likely forming the T6SS inner tube, which was shown to be required in a large copy number in canonical T6SS, e.g., up to ~1,000 molecules for a single *Vibrio cholerae* T6SS sheath-tube complex (Wang et al., 2017). As shown on Figure 4E and Supplementary Movies 1, 2, T6SS sheath dynamics was only partially restored when inducing IglC expression from pFNMB1 with 500 ng/ml of ATc. However, T6SS sheath dynamics was restored to the levels similar to the parental strain when using pFNMB2 for IglC expression (Figure 4F, Supplementary Movies 1, 2). Overall, this suggests that pFNMB plasmids are superior to the previously used plasmids for complementation in *F. novicida* and that pFNMB2 plasmid can be used to achieve high levels of protein expression.

**Figure 5:**
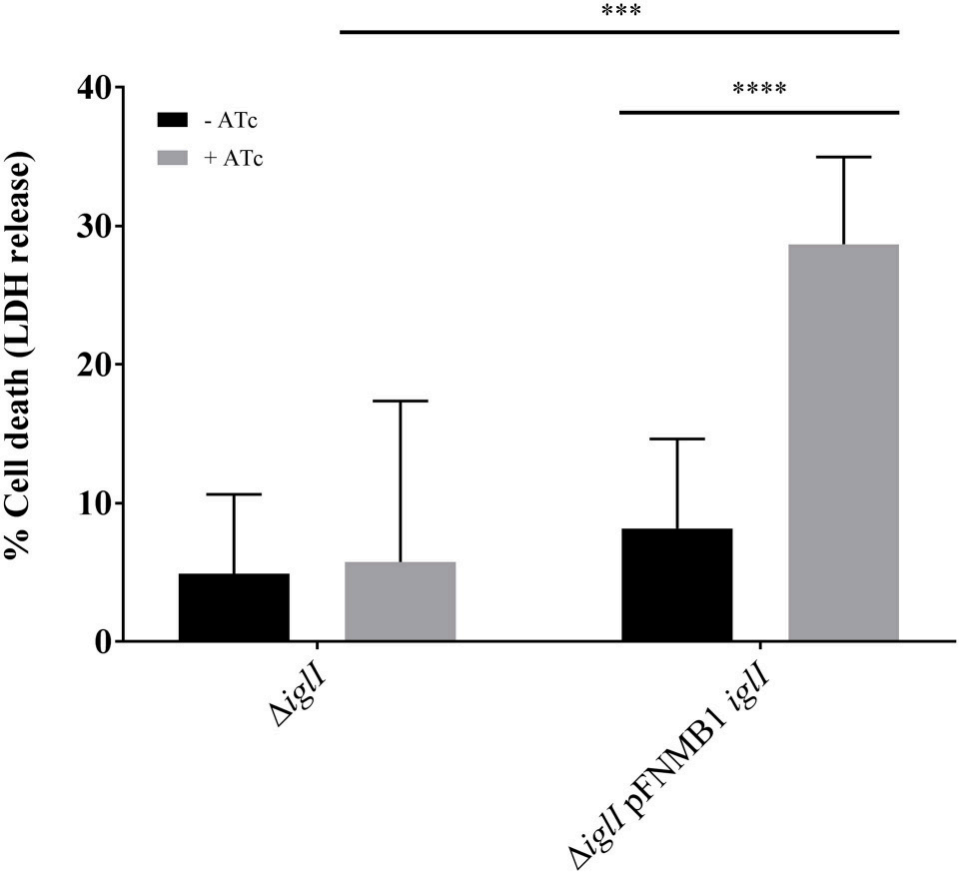
Complementation of Δ*iglI* mutant in BMDMs. Release of LDH from primed BMDMs supplemented with 0 and 1,000 ng/ml ATc 10 h after infection with *F. novicida* U112 *iglA-sfgfp* Δ*iglI* and *F. novicida* U112 *iglA-sfgfp* Δ*iglI* pFNMB1 *iglI*. Bacterial cultures were supplemented with 0 and 500 ng/ml ATc overnight. Black columns represent conditions without induction by ATc and gray columns represent conditions with ATc. Means and standard deviations of nine measurements originating from three biological replicates are shown. The two-tailed unpaired *t*-test with Welch's correction was used to test significant differences between two groups. ^***^P < 0.001 and ^****^P < 0.0001.

Intracellular *F. novicida* require a functional T6SS to escape from the phagosome in order to reach the replicative niche in the cytosol (Chong and Celli, 2010). Cytosolic *F. novicida* bacteria activate the absent in melanoma 2 (AIM2) inflammasome among other defense mechanisms, which leads to pyroptotic cell death and pro-inflammatory cytokine release (Fernandes-Alnemri et al., 2010; Jones et al., 2010). To test whether pFNMB1 can be used for complementation in bone marrow derived macrophages (BMDMs), we analyzed in-frame deletion mutant Δ*iglI* and the respective complemented strain for induction of pyroptosis in infected cells as a measure for phagosomal escape and thus T6SS function. We pre-induced expression of IglI from pFNMB1 with 0 and 500 ng/ml ATc overnight and then infected BMDMs, which were supplemented with 0 and 1,000 ng/ml ATc. After 10 h of infection, we observed significantly higher cell death for the complemented strain than for the in-frame deletion mutant without induced gene expression or for the in-frame deletion mutant without the plasmid (Figure 5). This result indicates that pFNMB1 can be used to restore T6SS activity in *F. novicida* mutant in BMDMs.

## Discussion

We generated stable mobilizable expression plasmids pFNMB1 and pFNMB2 for *F. novicida*. There are two major advantages using these plasmids. First, they can be easily mobilized from *E. coli* to *F. novicida*; second, they allow for inducible and homogeneous expression of inserted genes *in-vitro* and inside eukaryotic cells. We modified pKK289Km by insertion of the RP4 mobilization site as we experienced great difficulties transforming *F. novicida* by electroporation similarly to what was reported previously (Maier et al., 2004; LoVullo et al., 2006). The low electroporation efficiency in *F. novicida* is probably caused by the capsule and restriction-modification systems (Maier et al., 2004; LoVullo et al., 2006; Frank and Zahrt, 2007; Gallagher et al., 2008). Gallagher et al. (2008) suggested to first transform plasmid DNA into a *F. novicida* strain with all restriction-modification systems deleted and then use this isolated plasmid DNA to transform wild-type *F. novicida*. Importantly, the high efficiency of mobilization of the pFNMB plasmids can be reached without this step and therefore may allow for generation of large libraries of mutants and thus facilitate future screens and selections.

To express genes in a controlled manner, pFNMB1 and pFNMB2 contain a tetracycline inducible promoter system, which was used for *F. tularensis* (LoVullo et al., 2012). We could show that expression levels were dependent on ATc concentration in *F. novicida* (Figure 3D). In contrast to *F. tularensis* (LoVullo et al., 2012), we observed no growth defects of *F. novicida* in the presence of 1,000 ng/ml ATc. However, we noticed that the expression levels achieved from pKK289Km were higher than those from our constructs. One possible explanation for the lower induction levels of pFNMB1 compared to pKK289Km are the different RBS. However, pFNMB2 has a similar RBS as pKK289Km (except for the MluI restriction site); therefore, it is also possible that pFNMB2 is still partially repressed even at 1,000 ng/ml of ATc. This may suggest that cytosolic concentration of ATc reaches lower level in *F. novicida* than in *F. tularensis*. Indeed, differences in resistance levels toward tetracycline antibiotics and number of transporters were reported (Kingry and Petersen, 2014; Sutera et al., 2014). Additionally, in contrast to pKK289Km, the expression from pFNMB1 and pFNMB2 is homogenous throughout the bacterial population (Figures 3A–C). The reason for the heterogeneous gene expression from pKK289Km in *F. novicida* is unknown; however, spontaneous deletions or differential activation of the *gro*ESL promoter could be responsible.

Other suitable inducible promoter systems are difficult to use in *Francisella*. The *araBAD* promoter requires the uptake of L-arabinose for induction (Guzman et al., 1995); similarly the *lac* promoter requires lactose or isopropyl-β-D-thiogalactopyranosid (Polisky et al., 1976). Since *Francisella* lacks the L-arabinose and lactose degradation pathway (NCBI, RefSeq NC_008601.1, Larsson et al., 2005), it is questionable if these inducers are taken up. In addition, *Francisella* has a unique RNA polymerase composition with two different α subunits, which may interfere with promoter recognition of these commonly used inducible promoter systems subunits (Charity et al., 2007). A glucose repressible promoter system was described for *F. tularensis* (Horzempa et al., 2008), however, since glucose is a common carbon source, the use of such repressor could be problematic. In addition, a temperature dependent promoter was constructed for *F. tularensis* (Maier et al., 2004). However, since *Francisella* is an intracellular pathogen (Chong and Celli, 2010), many cell culture infections or *in vivo* experiments are performed at defined temperature and temperature shifting is impossible. Overall, the tetracycline inducible promoter system is likely the best option for *F. novicida* despite the apparent suboptimal level of derepression by ATc. Importantly, the possibility of inducing gene expression in cell culture or *in vivo* is a crucial advantage for testing the role of expressed genes during the pathogenesis of *F. novicida*.

In summary, we show that pFNMB1 and pFNMB2 are easy to mobilize into *F. novicida* and are stably maintained in the population. The tetracycline inducible promoter system is functional in *F. novicida* and can be used to tune gene expression levels. pFNMB1 and pFNMB2 exhibit homogeneous expression patterns in a population and can be used to complement chromosomal in-frame deletions. Overall, pFNMB1 and pFNMB2 may serve as useful tools for future studies of *F. novicida*.

## Author contributions

MBr and MBa designed experiments, analyzed, and interpreted the results. MBr generated strains and acquired all data except for the BMDM infection experiments. PB and RH designed, analyzed, and interpreted the BMDM infection experiments. RH acquired the data for the BMDM infection experiments. MBr and MBa wrote the manuscript. All authors read and approved the manuscript.

### Conflict of interest statement

The authors declare that the research was conducted in the absence of any commercial or financial relationships that could be construed as a potential conflict of interest.

## References

[B1] Abd H. Johansson T. Golovliov I. Sandström G. Forsman M. (2003). Survival and growth of *Francisella tularensis* in *Acanthamoeba castellanii*. Appl. Environ. Microbiol. 69, 600–606. 10.1128/AEM.69.1.600-606.2003 12514047 PMC152416

[B2] Anthony L. S. Gu M. Z. Cowley S. C. Leung W. W. Nano F. E. (1991). Transformation and allelic replacement in *Francisella* spp. J. Gen. Microbiol. 137, 2697–2703. 10.1099/00221287-137-12-2697 1791425

[B3] Ark N. M. Mann B. J. (2011). Impact of *Francisella tularensis* pilin homologs on pilus formation and virulence. Microb. Pathog. 51, 110–120. 10.1016/j.micpath.2011.05.001 21605655 PMC3120926

[B4] Basler M. Ho B. T. Mekalanos J. J. (2013). Tit-for-tat: type VI secretion system counterattack during bacterial cell-cell interactions. Cell 152, 884–894. 10.1016/j.cell.2013.01.042 23415234 PMC3616380

[B5] Bertram R. Hillen W. (2008). The application of Tet repressor in prokaryotic gene regulation and expression. Microb. Biotechnol. 1, 2–16. 10.1111/j.1751-7915.2007.00001.x 21261817 PMC3864427

[B6] Bönquist L. Lindgren H. Golovliov I. Guina T. Sjöstedt A. (2008). MglA and Igl proteins contribute to the modulation of *Francisella tularensis* live vaccine strain-containing phagosomes in murine macrophages. Infect. Immun. 76, 3502–3510. 10.1128/IAI.00226-08 18474647 PMC2493230

[B7] Brodmann M. Dreier R. F. Broz P. Basler M. (2017). *Francisella* requires dynamic type VI secretion system and ClpB to deliver effectors for phagosomal escape. Nat. Commun. 8:15853. 10.1038/ncomms15853 28621333 PMC5481754

[B8] Bröms J. E. Sjöstedt A. Lavander M. (2010). The role of the *Francisella tularensis* pathogenicity island in type VI secretion, intracellular survival, and modulation of host cell signaling. Front. Microbiol. 1:136. 10.3389/fmicb.2010.00136 21687753 PMC3109350

[B9] Charity J. C. Costante-Hamm M. M. Balon E. L. Boyd D. H. Rubin E. J. Dove S. L. (2007). Twin RNA polymerase-associated proteins control virulence gene expression in *Francisella tularensis*. PLoS Pathog. 3:e84. 10.1371/journal.ppat.0030084 17571921 PMC1891329

[B10] Chong A. Celli J. (2010). The *francisella* intracellular life cycle: toward molecular mechanisms of intracellular survival and proliferation. Front. Microbiol. 1:138. 10.3389/fmicb.2010.00138 21687806 PMC3109316

[B11] Clemens D. L. Ge P. Lee B. Y. Horwitz M. A. Zhou Z. H. (2015). Atomic structure of T6SS reveals interlaced array essential to function. Cell 160, 940–951. 10.1016/j.cell.2015.02.005 25723168 PMC4351867

[B12] de Bruin O. M. Duplantis B. N. Ludu J. S. Hare R. F. Nix E. B. Schmerk C. L. (2011). The biochemical properties of the *Francisella* pathogenicity island (FPI)-encoded proteins IglA, IglB, IglC, PdpB and DotU suggest roles in type VI secretion. Microbiology 157, 3483–3491. 10.1099/mic.0.052308-0 21980115 PMC3352279

[B13] de Bruin O. M. Ludu J. S. Nano F. E. (2007). The *Francisella* pathogenicity island protein IglA localizes to the bacterial cytoplasm and is needed for intracellular growth. BMC Microbiol. 7:1. 10.1186/1471-2180-7-1 17233889 PMC1794414

[B14] Eshraghi A. Kim J. Walls A. C. Ledvina H. E. Miller C. N. Ramsey K. M.. (2016). Secreted effectors encoded within and outside of the *Francisella* pathogenicity island promote intramacrophage growth. Cell Host Microbe 20, 573–583. 10.1016/j.chom.2016.10.008 27832588 PMC5384264

[B15] Fernandes-Alnemri T. Yu J. W. Juliana C. Solorzano L. Kang S. Wu J.. (2010). The AIM2 inflammasome is critical for innate immunity to *Francisella tularensis*. Nat. Immunol. 11, 385–393. 10.1038/ni.1859 20351693 PMC3111085

[B16] Frank D. W. Zahrt T. C. (2007). Genetics and genetic manipulation in *Francisella tularensis*. Ann. N.Y. Acad. Sci. 1105, 67–97. 10.1196/annals.1409.008 17395725

[B17] Gallagher L. A. McKevitt M. Ramage E. R. Manoil C. (2008). Genetic dissection of the *Francisella novicida* restriction barrier. J. Bacteriol. 190, 7830–7837. 10.1128/JB.01188-08 18835994 PMC2583604

[B18] Gil H. Platz G. J. Forestal C. A. Monfett M. Bakshi C. S. Sellati T. J.. (2006). Deletion of TolC orthologs in *Francisella tularensis* identifies roles in multidrug resistance and virulence. Proc. Natl. Acad. Sci. U.S.A. 103, 12897–12902. 10.1073/pnas.0602582103 16908853 PMC1568944

[B19] Golovliov I. Sjöstedt A. Mokrievich A. Pavlov V. (2003). A method for allelic replacement in *Francisella tularensis*. FEMS Microbiol. Lett. 222, 273–280. 10.1016/S0378-1097(03)00313-6 12770718

[B20] Gossen M. Bujard H. (1992). Tight control of gene expression in mammalian cells by tetracycline-responsive promoters. Proc. Natl. Acad. Sci. U.S.A. 89, 5547–5551. 10.1073/pnas.89.12.5547 1319065 PMC49329

[B21] Guzman L. M. Belin D. Carson M. J. Beckwith J. (1995). Tight regulation, modulation, and high-level expression by vectors containing the arabinose PBAD promoter. J. Bacteriol. 177, 4121–4130. 10.1128/jb.177.14.4121-4130.1995 7608087 PMC177145

[B22] Haase J. Lurz R. Grahn A. M. Bamford D. H. Lanka E. (1995). Bacterial conjugation mediated by plasmid RP4: RSF1010 mobilization, donor-specific phage propagation, and pilus production require the same Tra2 core components of a proposed DNA transport complex. J. Bacteriol. 177, 4779–4791. 10.1128/jb.177.16.4779-4791.1995 7642506 PMC177245

[B23] Harms A. Segers F. H. Quebatte M. Mistl C. Manfredi P. Körner J.. (2017). Evolutionary dynamics of pathoadaptation revealed by three independent acquisitions of the VirB/D4 type IV secretion system in *Bartonella*. Genome Biol. Evol. 9, 761–776. 10.1093/gbe/evx042 28338931 PMC5381568

[B24] Horzempa J. Tarwacki D. M. Carlson P. E. Robinson C. M. Nau G. J. (2008). Characterization and application of a glucose-repressible promoter in *Francisella tularensis*. Appl. Environ. Microbiol. 74, 2161–2170. 10.1128/AEM.02360-07 18245238 PMC2292608

[B25] Jones J. W. Kayagaki N. Broz P. Henry T. Newton K. O'Rourke K.. (2010). Absent in melanoma 2 is required for innate immune recognition of *Francisella tularensis*. Proc. Natl. Acad. Sci. U.S.A. 107, 9771–9776. 10.1073/pnas.1003738107 20457908 PMC2906881

[B26] Kingry L. C. Petersen J. M. (2014). Comparative review of *Francisella tularensis* and *Francisella novicida*. Front. Cell. Infect. Microbiol. 4:35. 10.3389/fcimb.2014.00035 24660164 PMC3952080

[B27] Kudryashev M. Wang R. Y. R. Brackmann M. Scherer S. Maier T. Baker D.. (2015). Structure of the type VI secretion system contractile sheath. Cell 160, 952–962. 10.1016/j.cell.2015.01.037 25723169 PMC4359589

[B28] Lai X. H. Golovliov I. Sjöstedt A. (2004). Expression of IglC is necessary for intracellular growth and induction of apoptosis in murine macrophages by *Francisella tularensis*. Microb. Pathog. 37, 225–230. 10.1016/j.micpath.2004.07.002 15519043

[B29] Larsson P. Oyston P. C. F. Chain P. Chu M. C. Duffield M. Fuxelius H. H.. (2005). The complete genome sequence of *Francisella tularensis*, the causative agent of tularemia. Nat. Genet. 37, 153–159. 10.1038/ng1499 15640799

[B30] Lindemann S. R. Peng K. Long M. E. Hunt J. R. Apicella M. A. Monack D. M.. (2011). *Francisella tularensis* Schu S4 O-antigen and capsule biosynthesis gene mutants induce early cell death in human macrophages. Infect. Immun. 79, 581–594. 10.1128/IAI.00863-10 21078861 PMC3028865

[B31] Lindgren H. Shen H. Zingmark C. Golovliov I. Conlan W. Sjöstedt A. (2007). Resistance of *Francisella tularensis* strains against reactive nitrogen and oxygen species with special reference to the role of KatG. Infect. Immun. 75, 1303–1309. 10.1128/IAI.01717-06 17210667 PMC1828546

[B32] Lindgren M. Bröms J. E. Meyer L. Golovliov I. Sjöstedt A. (2013). The *Francisella tularensis* LVS Δ*pdpC* mutant exhibits a unique phenotype during intracellular infection. BMC Microbiol. 13:20. 10.1186/1471-2180-13-20 23356941 PMC3562505

[B33] LoVullo E. D. Miller C. N. Pavelka M. S. Kawula T. H. (2012). TetR-based gene regulation systems for *Francisella tularensis*. Appl. Environ. Microbiol. 78, 6883–6889. 10.1128/AEM.01679-12 22820330 PMC3457491

[B34] LoVullo E. D. Sherrill L. A. Pavelka M. S. (2009). Improved shuttle vectors for *Francisella tularensis* genetics. FEMS Microbiol. Lett. 291, 95–102. 10.1111/j.1574-6968.2008.01440.x 19067747 PMC2704062

[B35] LoVullo E. D. Sherrill L. A. Perez L. L. Pavelka M. S. (2006). Genetic tools for highly pathogenic *Francisella tularensis* subsp. tularensis. Microbiology 152, 3425–3435. 10.1099/mic.0.29121-0 17074911

[B36] Maier T. M. Havig A. Casey M. Nano F. E. Frank D. W. Zahrt T. C. (2004). Construction and characterization of a highly efficient *Francisella* shuttle plasmid. Appl. Environ. Microbiol. 70, 7511–7519. 10.1128/AEM.70.12.7511-7519.2004 15574954 PMC535190

[B37] Maier T. M. Pechous R. Casey M. Zahrt T. C. Frank D. W. (2006). *In vivo* Himar1-based transposon mutagenesis of *Francisella tularensis*. Appl. Environ. Microbiol. 72, 1878–1885. 10.1128/AEM.72.3.1878-1885.2006 16517634 PMC1393221

[B38] Nano F. E. Schmerk C. (2007). The *Francisella* pathogenicity island. Ann. N.Y. Acad. Sci. 1105, 122–137. 10.1196/annals.1409.000 17395722

[B39] Norqvist A. Kuoppa K. Sandström G. (1996). Construction of a shuttle vector for use in *Francisella tularensis*. FEMS Immunol. Med. Microbiol. 13, 257–260. 10.1111/j.1574-695X.1996.tb00248.x 8861040

[B40] Oyston P. C. F. Sjostedt A. Titball R. W. (2004). Tularaemia: bioterrorism defence renews interest in *Francisella tularensis*. Nat. Rev. Microbiol. 2, 967–978. 10.1038/nrmicro1045 15550942

[B41] Pavlov V. M. Mokrievich A. N. Volkovoy K. (1996). Cryptic plasmid pFNL10 from *Francisella novicida*-like F6168: the base of plasmid vectors for *Francisella tularensis*. FEMS Immunol. Med. Microbiol. 13, 253–256. 10.1111/j.1574-695X.1996.tb00247.x 8861039

[B42] Polisky B. Bishop R. J. Gelfand D. H. (1976). A plasmid cloning vehicle allowing regulated expression of eukaryotic DNA in bacteria. Proc. Natl. Acad. Sci. U.S.A. 73, 3900–3904. 10.1073/pnas.73.11.3900 1069275 PMC431258

[B43] Pomerantsev A. P. Golovliov I. R. Ohara Y. Mokrievich A. N. Obuchi M. Norqvist A.. (2001a). Genetic organization of the *Francisella* plasmid pFNL10. Plasmid 46, 210–222. 10.1006/plas.2001.1548 11735370

[B44] Pomerantsev A. P. Obuchi M. Ohara Y. (2001b). Nucleotide sequence, structural organization, and functional characterization of the small recombinant plasmid pOM1 that is specific for *Francisella tularensis*. Plasmid 46, 86–94. 10.1006/plas.2001.1538 11591134

[B45] Rasko D. A. Esteban C. D. Sperandio V. (2007). Development of novel plasmid vectors and a promoter trap system in *Francisella tularensis* compatible with the pFLN10 based plasmids. Plasmid 58, 159–166. 10.1016/j.plasmid.2007.03.002 17459476 PMC2013926

[B46] Santic M. Molmeret M. Barker J. R. Klose K. E. Dekanic A. Doric M.. (2007). A *Francisella tularensis* pathogenicity island protein essential for bacterial proliferation within the host cell cytosol. Cell. Microbiol. 9, 2391–2403. 10.1111/j.1462-5822.2007.00968.x 17517064

[B47] Santic M. Ozanic M. Semic V. Pavokovic G. Mrvcic V. Kwaik Y. A. (2011). Intra-vacuolar proliferation of *F. Novicida* within *H. Vermiformis*. Front. Microbiol. 2:78. 10.3389/fmicb.2011.00078 21747796 PMC3128938

[B48] Schindelin J. Arganda-Carreras I. Frise E. Kaynig V. Longair M. Pietzsch T.. (2012). Fiji: an open-source platform for biological-image analysis. Nat. Methods 9, 676–682. 10.1038/nmeth.2019 22743772 PMC3855844

[B49] Schmidt M. Klimentova J. Rehulka P. Straskova A. Spidlova P. Szotakova B.. (2013). *Francisella tularensis* subsp. holarctica DsbA homologue: a thioredoxin-like protein with chaperone function. Microbiol. Read. Engl. 159, 2364–2374. 10.1099/mic.0.070516-0 24014665

[B50] Scholz O. Henssler E. M. Bail J. Schubert P. Bogdanska-Urbaniak J. Sopp S.. (2004). Activity reversal of Tet repressor caused by single amino acid exchanges. Mol. Microbiol. 53, 777–789. 10.1111/j.1365-2958.2004.04159.x 15255892

[B51] Sutera V. Levert M. Burmeister W. P. Schneider D. Maurin M. (2014). Evolution toward high-level fluoroquinolone resistance in *Francisella* species. J. Antimicrob. Chemother. 69, 101–110. 10.1093/jac/dkt321 23963236

[B52] Tempel R. Lai X. H. Crosa L. Kozlowicz B. Heffron F. (2006). Attenuated *Francisella novicida* transposon mutants protect mice against wild-type challenge. Infect. Immun. 74, 5095–5105. 10.1128/IAI.00598-06 16926401 PMC1594869

[B53] Vettiger A. Basler M. (2016). Type VI secretion system substrates are transferred and reused among sister cells. Cell 167, 99–110.e12. 10.1016/j.cell.2016.08.023 27616061

[B54] Wang J. Brackmann M. Castaño-Díez D. Kudryashev M. Goldie K. N. Maier T.. (2017). Cryo-EM structure of the extended type VI secretion system sheath-tube complex. Nat. Microbiol. 2, 1507–1512. 10.1038/s41564-017-0020-7 28947741

[B55] Weiss D. S. Brotcke A. Henry T. Margolis J. J. Chan K. Monack D. M. (2007). *In vivo* negative selection screen identifies genes required for *Francisella* virulence. Proc. Natl. Acad. Sci. U.S.A. 104, 6037–6042. 10.1073/pnas.0609675104 17389372 PMC1832217

[B56] Zogaj X. Klose K. E. (2010). Genetic manipulation of *francisella tularensis*. Front. Microbiol. 1:142. 10.3389/fmicb.2010.00142 21607086 PMC3095392

