# Supplementary material for: Mobilizable Plasmids for Tunable Gene Expression in Francisella novicida
Source: Front Cell Infect Microbiol. 2018 Aug 31;8:284. doi: 10.3389/fcimb.2018.00284 (PMC6128221; doi:10.3389/fcimb.2018.00284)
Supplement: Supplementary file 1 [file Table_1.docx]

***Supplementary Material***

**Mobilizable Plasmids for Tunable Gene Expression in *Francisella novicida*.**

Maj Brodmann^1^, Rosalie Heilig^2^, Petr Broz^2^ and Marek Basler^1*^

^1^ Biozentrum, University of Basel, Basel, Switzerland

^2^ Department of Biochemistry, University of Lausanne, Épalinges, Switzerland

^*^ **Correspondence:**

Dr. Marek Basler
[marek.basler@unibas.ch](mailto:marek.basler@unibas.ch)

1. **Supplementary tables**

**Table S1: Strains used in this study, related to Material and Methods**

| **Organism** | **Genotype** | **Plasmid** | **Relevant features** | **Source** |
| --- | --- | --- | --- | --- |
| *Francisella novicida* U112 | *iglA-sfgfp* |  | Parental strain, C‐terminal chromosomal fusion of *sfgfp* to *iglA* | (Clemens et al., 2015) |
|  | *iglA-sfgfp ΔiglF* |  | Deletion of *iglF* | (Brodmann et al., 2017) |
|  | *iglA-sfgfp ΔiglI* |  | Deletion of *iglI* | (Brodmann et al., 2017) |
|  | *iglA-sfgfp ΔiglC* |  | Deletion of *iglC* | This study |
|  |  | pKK289Km *gfp* | Constitutive expression of GFP under *groESL* promoter | (Bönquist et al., 2008) |
|  | *iglA-sfgfp ΔiglF* | pKK289Km *iglF* | Constitutive complementation *iglF* | This study |
|  | *iglA-sfgfp ΔiglI* | pKK289Km *iglI* | Constitutive complementation *iglI* | This study |
|  |  | pFNMB1 *msfgfp* | RP4 mobilization site, ATC inducible expression of *msfgfp*, *iglC* RBS | This study |
|  |  | pFNMB2 *msfgfp* | RP4 mobilization site, ATC inducible expression of *msfgfp*, pKK289Km RBS | This study |
|  | *iglA-sfgfp ΔiglF* | pFNMB1 *iglF* | Inducible expression of *iglF* | This study |
|  | *iglA-sfgfp ΔiglI* | pFNMB1 *iglI* | Inducible expression of *iglI* | This study |
|  | *iglA-sfgfp ΔiglC* | pFNMB1 *iglC* | Inducible expression of *iglC* | This study |
|  | *iglA-sfgfp ΔiglC* | pFNMB2 *iglC* | Inducible expression of *iglC* | This study |
| *Escherichia coli* DH5α *λpir* |  | pFNMB1 *msfgfp* | RP4 mobilization site, ATC inducible expression of *msfgfp*, *iglC* RBS | This study |
|  |  | pFNMB2 *msfgfp* | RP4 mobilization site, ATC inducible expression of *msfgfp*, pKK289Km RBS | This study |

**Table S2: Plasmids and primers used to generate plasmids pFNMB1 and pFNMB2, related to Material and Methods**

| **Plasmid Name** | **Feature** | **Primers used** | | **Restriction sites** | **Source** |
| --- | --- | --- | --- | --- | --- |
| pKK289Km *gfp* | Backbone |  |  |  | (Bönquist et al., 2008) |
| pDMK3 | RP4 mobilization site | oriT-long_EcoR1.FOR | TCAGTAGAATTCTTAGATCCAGCCGACCAG | EcoRI | (Lindgren et al., 2007) |
|  |  | oriT-long_Sbf1.REV | TCAGTACCTGCAGGTCAATCCTTTTTGTCCGGTG | SbfI |  |
| pDMK3 | MCS | Tet_MCS_2.FOR | CAGTGATAGAGAGTCGACCCTCGAGTACGCGTCTCTAG |  | (Lindgren et al., 2007) |
|  |  | Tet_MCS_Term_2.REV | CAGACCGCCCCGGGAGAGCTCAGGT |  |  |
| pEDL17 | ATC inducible promoter cassette | Tet_MCS_Spe1.For | TCAGTAACTAGTTTAAGACCCACTTTCACATT | SpeI | (LoVullo et al., 2012) |
|  |  | Tet_MCS_1.REV | CTCGAGGGTCGACTCTCTATCACTGATAGGGACAAGTCTAGATATTGAG |  |  |
| pBAD24 | *rrnB*  T1 and T2 terminators | Tet_MCS_Term_3.FOR | CTCTCCCGGGGCGGTCTGATAAAACAGAATTTGCCT | EcoRI | (Guzman et al., 1995) |
|  |  | Tet_MCS_Term_3_EcoR1.REV | TCAGTAGAATTCAAAAGGCCATCCGTCAGGAT |  |  |

**Table S3: Plasmids and primers used to generate mutants, related to Material and Methods**

| **Plasmid Name** | **Peptide scar left on the**  **chromosome after allelic**  **exchange** | **Primers used** | |
| --- | --- | --- | --- |
| pDMK3 Δ*iglC* | MSEMITRQQVVAKIGYIAAA* | dFTN_1322_1_Xho1.FOR | TCAGTACTCGAGGTTGAATTAAGCTGTAAATATCAT |
|  |  | dFTN_1322_1.REV | ACAACAGGTAGTTGCTAAAATAGGATATATTG |
|  |  | dFTN_1322_2.FOR | TTTTAGCAACTACCTGTTGTCTTGTTATCAT |
|  |  | dFTN_1322_2_Not1.REV | TCAGTAGCGGCCGCTCCAGTTCAGTATAAACTTATG |
|  |  | dFTN_1323_Det.FOR | ACCTGTCTGCAAACTTTCAACA |
|  |  | dFTN_1322_Det.REV | GAAACCTTGATGTTGCTGCA |
| pKK289Km *iglF* |  | FTN_1313_Nde1.FOR | TCAGTACATATGAATAATGATATTGATAAATGGTTTGAA |
|  |  | FTN_1313_Sac1_REV | TCAGTAGAGCTCTTAAATTTTCCAATAAGCTTCTTG |
|  |  | pKK289Km_Seq1.FOR | CCCCAAACATCGCAAAAGGT |
|  |  | pKK289Km_Seq1.REV | CACGCCACATCTTGCGAATA |
| pKK289Km *iglI* |  | FTN_1317_Nde1.FOR | TCAGTACATATGAGTCAGATAATATCTACACTAAATAAT |
|  |  | FTN_1317_EcoR1.REV | TCAGTAGAATTCTATATGTCAAAAAGATCTTCAAAATAGT |
|  |  | pKK289Km_Seq1.FOR | CCCCAAACATCGCAAAAGGT |
|  |  | pKK289Km_Seq1.REV | CACGCCACATCTTGCGAATA |
| pFNMB1 *msfgfp* |  | iglC_RBS_Mlu1_GFP.FOR | TCAGTACTCGAGAGGAGAACGCGTATGGGATCTAAAGGTGAAGAACT |
|  |  | msfGFP_Xma_REV | TCAGTACCCGGGTTATTTGTAGAGCTCATCCATG |
|  |  | pFNMB_seq_FOR | TCATAGAAGCTTGCATGCCTG |
|  |  | pFNMB_seq_REV | GAGACCCCACACTACCATCG |
| pFNMB2 *msfgfp* |  | Tet_MCS_Spe1.For | TCAGTAACTAGTTTAAGACCCACTTTCACATT |
|  |  | Tet_pKK289Km_RBS_Mlu1_gfp_1.REV | ACGCGTATCTCCTTCTTAAATCTGCAGTCTCTATCACTGATAGGGACAAG |
|  |  | Tet_pKK289Km_RBS_Mlu1_gfp_2.FOR | ATTTAAGAAGGAGATACGCGTATGGGATCTAAAGGTGAAGAACTGTTCAC |
|  |  | msfGFP_Xma_REV | TCAGTACCCGGGTTATTTGTAGAGCTCATCCATG |
|  |  | pFNMB_seq_FOR | TCATAGAAGCTTGCATGCCTG |
|  |  | pFNMB_seq_REV | GAGACCCCACACTACCATCG |
| pFNMB1 *iglF* |  | FTN_1313_Mlu1.FOR | TCAGTAACGCGTATGAATAATGATATTGATAAATGGTTTGAA |
|  |  | FTN_1313_Sac1_REV | TCAGTAGAGCTCTTAAATTTTCCAATAAGCTTCTTG |
|  |  | pFNMB_seq_FOR | TCATAGAAGCTTGCATGCCTG |
|  |  | pFNMB_seq_REV | GAGACCCCACACTACCATCG |
| pFNMB1 *iglI* |  | FTN_1317_Mlu1.FOR | TCAGTAACGCGTATATGAGTCAGATAATATCTACACTAAATAAT |
|  |  | FTN_1317_Sac1.REV | TCAGTAGAGCTCTTATATGTCAAAAAGATCTTCAAAATAGT |
|  |  | pFNMB_seq_FOR | TCATAGAAGCTTGCATGCCTG |
|  |  | pFNMB_seq_REV | GAGACCCCACACTACCATCG |
| pFNMB1 *iglC* |  | FTN_1322_Mlu1.FOR | TCAGTAACGCGTATGAGTGAGATGATAACAAG |
|  |  | FTN_1322_Sac1.REV | TCAGTAGAGCTCCTATGCAGCTGCAATATATC |
|  |  | pFNMB_seq_FOR | TCATAGAAGCTTGCATGCCTG |
|  |  | pFNMB_seq_REV | GAGACCCCACACTACCATCG |
| pFNMB2 *iglC* |  | FTN_1322_Mlu1.FOR | TCAGTAACGCGTATGAGTGAGATGATAACAAG |
|  |  | FTN_1322_Sac1.REV | TCAGTAGAGCTCCTATGCAGCTGCAATATATC |
|  |  | pFNMB_seq_FOR | TCATAGAAGCTTGCATGCCTG |
|  |  | pFNMB_seq_REV | GAGACCCCACACTACCATCG |

1. **Supplementary movies**

**Supplementary movie 1: T6SS sheath dynamics in complemented mutants.** IglA-sfGFP was monitored in *F. novicida* U112 *iglA-sfgfp* Δ*iglF* pKK289Km *iglF,* *F. novicida* U112 *iglA-sfgfp* Δ*iglF* pFNMB1 *iglF*, *F. novicida* U112 *iglA-sfgfp* Δ*iglI* pKK289Km *iglI*, *F. novicida* U112 *iglA-sfgfp* Δ*iglI* pFNMB1 *iglI, F. novicida* U112 *iglA-sfgfp* Δ*iglC* pFNMB1 *iglC* and *F. novicida* U112 *iglA-sfgfp* Δ*iglC* pFNMB2 *iglC*. Gene expression was induced with 250 ng/ml of ATc except for IglC, which was induced with 500 ng/ml of ATc. The bacteria were imaged for 5 min at a frame rate of 2 frames per minutes and for each strain two representative time-lapse image series are shown. The movie consists of the GFP channel and the scale bar represents 1 µm. Fields of view are 3.3 x 3.3 µm. Movies play at a frame rate of 5 frames per second.

**Supplementary movie 2: Overview of T6SS sheath dynamics in complemented mutants.** IglA-sfGFP was monitored in *F. novicida* U112 *iglA-sfgfp* Δ*iglF* pKK289Km *iglF,* *F. novicida* U112 *iglA-sfgfp* Δ*iglF* pFNMB1 *iglF*, *F. novicida* U112 *iglA-sfgfp* Δ*iglI* pKK289Km *iglI*, *F. novicida* U112 *iglA-sfgfp* Δ*iglI* pFNMB1 *iglI, F. novicida* U112 *iglA-sfgfp* Δ*iglC* pFNMB1 *iglC* and *F. novicida* U112 *iglA-sfgfp* Δ*iglC* pFNMB2 *iglC*. Gene expression was induced with 250 ng/ml of ATc except for IglC, which was induced with 500 ng/ml of ATc. The bacteria were imaged for 5 min at a frame rate of 2 frames per minutes and for each strain two representative time-lapse image series are shown. The movie consists of the GFP channel and the scale bar represents 5 µm. Fields of view are 39 x 26 µm. Movies play at a frame rate of 5 frames per second.

1. **Supplementary references**

Bönquist, L., Lindgren, H., Golovliov, I., Guina, T., and Sjöstedt, A. (2008). MglA and Igl proteins contribute to the modulation of *Francisella tularensis* live vaccine strain-containing phagosomes in murine macrophages. *Infect. Immun.* *76*, 3502–3510.

Brodmann, M., Dreier, R.F., Broz, P., and Basler, M. (2017). *Francisella* requires dynamic type VI secretion system and ClpB to deliver effectors for phagosomal escape. *Nat. Commun. 8*, 15853.

Clemens, D.L., Ge, P., Lee, B.-Y., Horwitz, M.A., and Zhou, Z.H. (2015). Atomic Structure of T6SS Reveals Interlaced Array Essential to Function. *Cell* *160*, 940–951.

Guzman, L.M., Belin, D., Carson, M.J., and Beckwith, J. (1995). Tight regulation, modulation, and high-level expression by vectors containing the arabinose PBAD promoter. *J. Bacteriol.* *177*, 4121–4130.

Lindgren, H., Shen, H., Zingmark, C., Golovliov, I., Conlan, W., and Sjöstedt, A. (2007). Resistance of *Francisella tularensis* strains against reactive nitrogen and oxygen species with special reference to the role of KatG. *Infect. Immun.* *75*, 1303–1309.

LoVullo, E.D., Miller, C.N., Pavelka, M.S., and Kawula, T.H. (2012). TetR-based gene regulation systems for *Francisella tularensis*. *Appl. Environ. Microbiol.* *78*, 6883–6889.
